# Are continuous locking headless bone screws with locking plate fixation effective for bridging fracture gaps in far cortical locking constructs? An in vitro biomechanical and finite element study under simplified axial loading

**DOI:** 10.3389/fbioe.2026.1736372

**Published:** 2026-03-16

**Authors:** Chen-Chiang Lin, Ya-Han Chan, Po-Feng Su

**Affiliations:** 1 Department of Orthopedics, National Taiwan University Hospital Yun-Lin Branch, Douliou, Yunlin, Taiwan; 2 Department of Biomedical Engineering, National Yang Ming Chaio Tung University, Hsinchu, Taiwan; 3 Department of Orthopedics, Changhua Christian Hospital, Changhua, Taiwan

**Keywords:** biomechanical test, bone fracture, far cortical locking constructs, headless bone screw, locking plate fixation

## Abstract

**Introduction:**

Locking screws typically provide more stable fixation than conventional cortical screws. However, the rigid connection between the locking screw head and plate hole can cause stress concentrations and lead to early failure under cyclic loading. This study aimed to compare the mechanical stability of a continuous (headless) locking screw with standard locking screws and cortical screws, either in a standard (flush with the bone) or in far cortical locking constructs, using biomechanical testing and finite element analysis (FEA).

**Methods:**

The Trident Distal Radial Locking Plate (A-Plus Biotechnology Co., Ltd., Taiwan) was used to repair a simulated distal radial diaphysis fracture and was secured to the bone using different screw types. The fracture gap was bridged with the plate placed flush with the bone and with the plate offset from the bone surface by 4 mm (far cortical locking (FCL)). The constructs were subjected to static compression and cyclic axial loading tests to assess construct stiffness and yield load, as well as endurance and failure mechanisms under cyclic loading, while FEA was used to evaluate the von Mises stress distribution on the plates and screws.

**Results:**

The Group 3a (flush with bone, continuous locking screws) had a significantly higher yield load than Groups 2b (FCL with locking screws) and 3b (FCL with continuous locking screws) (p < 0.05). In addition, Group 1 (flush with bone, cortical screws) was found to be significantly stiffer than Groups 2b and 3b (p < 0.01). For the dynamic testing, Group 3a showed intermediate endurance and failed predominantly by screw pull-out, whereas Groups 1 and 2 primarily failed by plate deformation. The FEA results showed stress concentrations at the screw neck of the standard locking screws and around the threaded regions of the continuous locking screws.

**Conclusion:**

Continuous locking screws placed flush with the bone had the greatest fixation strength of all configurations tested. However, using continuous locking screws in an FCL configuration may redirect stress to the threaded region of the screw neck, potentially increasing the risk of failure.

## Introduction

Locking plates are widely utilized in the fixation of bone fractures, and the screw holes can typically accommodate both locking or non-locking screws. Locking screws generally offer more stable fracture fixation than conventional non-locking screws. However, the rigid connection formed between the head of the locking screw and the plate hole in both standard (flush with the bone) and far cortical locking (FCL) constructs bridges the force transfer from the bone to the plate (Bottlang et al., 2009), which can lead to fracturing of the screw head or shaft under cyclic loading (Iwata et al., 2017; Kankanalu et al., 2021; Raducha et al., 2022; Park et al., 2019). Postoperative failure of the screw can cause further instability and lead to complications with fracture healing (Panagiotopoulou et al., 2019).

**FIGURE 1:**
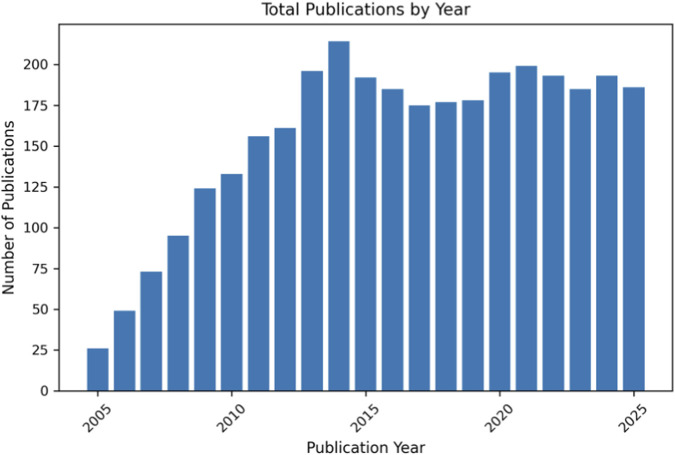
Annual number of publications related to bone plate fixation study from 2005 to 2025, illustrating a sustained increase in research output and scientific interest in fixation strategies. Data were obtained from PubMed (accessed on 2 February 2026) using the keywords bone plate and locking screw.

The incidence of screw failure has been reported at a rate of 4.2% after locking plate fixation in distal femoral fractures (Gurung et al., 2024). While the failure rate is low, the procedure to remove the broken screw or implant is challenging because forcefully removing the screws can cause additional damage to the bone or soft tissues (Park et al., 2019). The locking mechanism between the screw head and plate hole is designed to restrain relative movement between the surfaces of the bone and plate (Lin et al., 2025), but this rigidity can also cause stress concentrations to develop around the sharp transition from the screw shank to screw head, which puts the screw at greater risk of failure. Excessive construct stiffness and stress concentration in conventional locked plating have motivated the development of fixation concepts that introduce controlled flexibility while maintaining angular stability (Bottlang et al., 2009). Moreover, the temporal trend in publications related to bone plate fixation and locking screw constructs for bridging fracture gaps is illustrated in Figure 1, demonstrating a sustained increase in research activity and clinical interest in this field.

A continuous thread locking screw (A-Plus Biotechnology Co., Ltd., Taiwan) has been introduced with a thread profile that extends from the shank toward the screw head, reducing the geometric discontinuity typically present at the head–neck junction of conventional locking screws. Relative to the conventional locking screw used in this study, the continuous locking screw has a smaller head thread pitch (0.5 mm vs. 0.6 mm) and a reduced head taper angle (13° vs. 17°), while remaining compatible with standard locking plate insertion. Notably, this design does not employ a matched head–plate thread engagement and is not intended to introduce controlled construct flexibility (e.g., far cortical locking). The main purpose of this study was to compare whether the stability of locking plates secured with continuous locking screws via far cortical locking constructs is better than that of standard locking screws and cortical screws, using biomechanical testing and finite element models. Additional quantitative stress-concentration assessments are provided in Supplementary Appendix A.

## Materials and methods

### Mechanical tests

A Trident Distal Radial Locking Plate (A-Plus Biotechnology Co., Ltd., Taiwan) was used to repair a simulated distal radial diaphysis fracture. The screws were inserted either flush with the bone or with far cortical locking. The entire construct was subjected to axial compression loading and evaluated for stiffness, strength, and failure mode (Deng et al., 2022; Kritsaneephaiboon et al., 2025). Additionally, the model was placed under cyclic loading to determine if the failure mode was influenced by the type of construct used.

**FIGURE 2:**
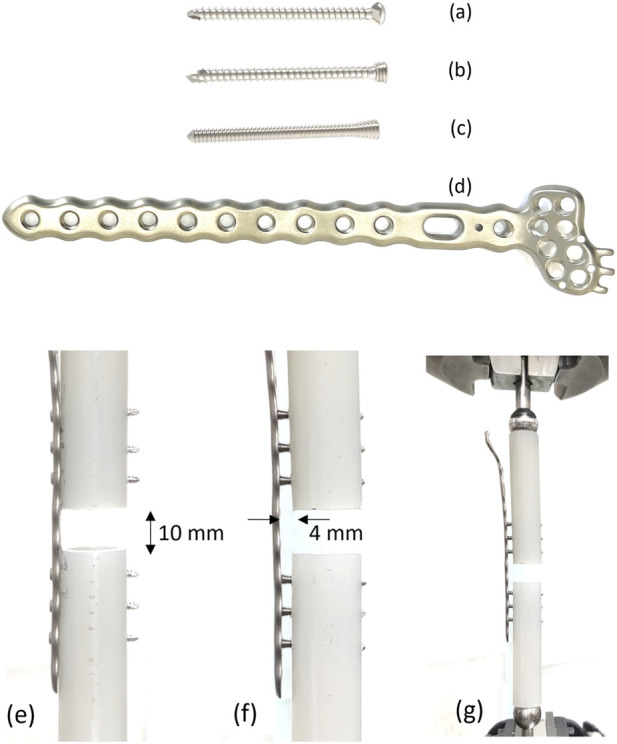
Three screw designs were tested: **(a)** cortical screw (2.4 mm diameter, 1.0 mm pitch), **(b)** locking screw (2.4 mm diameter, 1.0 mm pitch), **(c)** continuous locking screw (2.4 mm diameter, 0.6 mm pitch). **(d)** An 11-hole titanium TDRL Plate. **(e)** TDRL plate flush with the bone and fixed with screws; **(f)** TDRL plate with 4 mm offset from the cortical bone (FCL) and fixed with locking screws; **(g)** tested under static and dynamic axial compression using an MTS instrument.

Three different screw designs (A-Plus Biotechnology Co., Ltd., Taiwan) were evaluated in this study, all of which were 30 mm long. The first design was a cortical screw with a 2.4 mm diameter thread, 1.0 mm thread pitch, and a cylindrical profile. The second design was a locking screw, also with a 2.4 mm diameter thread, 1.0 mm thread pitch, and a cylindrical profile. The third design was a continuous locking screw, with a 2.4 mm diameter thread, a 0.6 mm thread pitch, and a cylindrical profile for the screw shaft (Figures 2a–c). An 11-hole Trident Distal Radial Locking Plate (TDRL Plate) was used as a bridging implant, with a thickness of 2 mm, width of 7.6 mm, and length of 126 mm. All implants were made from titanium alloy (Ti–6Al–4V), as shown in Figure 2d.

Five specimens were assigned to each test group. Group 1 consisted of a TDRL plate positioned flush with the bone and fixed with cortical screws (Figure 2e). Group 2a consisted of a TDRL plate flush with the bone and fixed with locking screws. Group 2b consisted of a TDRL plate offset by 4 mm from the surface of the cortical bone (termed ‘Far Cortical Locking’ (FCL)) and fixed with locking screws (Figure 2f). Group 3a consisted of a TDRL plate flush with the bone and fixed with continuous locking screws. Group 3b consisted of a TDRL plate with an FCL offset and fixed with continuous locking screws. There was no group arranged for cortical screws with an FCL bone plate because of the unrestricted freedom that would create between the screw head and plate. Table 1 details the groups for the mechanical tests.

**TABLE 1:** Test groups in the mechanical study, detailing the construction, positioning, and type of screws used for fixation in each group.

Group	Plate position	Screw type
Group 1	Flush with bone	Cortical screws
Group 2a	Flush with bone	Locking screws
Group 2b	Offset from cortical bone (4 mm)	Locking screws
Group 3a	Flush with bone	Continuous locking screws
Group 3b	Offset from cortical bone (4 mm)	Continuous locking screws

Synthetic bone materials are commonly used for mechanical testing as substitutes for human bone to minimize inter-specimen variability (Döbele et al., 2014). Hollow sawbones with a diameter of 20 mm and a wall thickness of 2 mm (Sawbones #3403-41; Pacific Research Laboratories, Vashon, Washington) were cut in half. TDLR plates were then secured to the sawbones with three screws in both the proximal and distal bone surrogates, creating a 10 mm osteotomy gap to replicate a comminuted fracture pattern (Yang et al., 2018). Each screw was tightened to 0.8 Nm using a torque limiter.

A total of 5 static and 3 dynamic compression tests were performed on each of the 5 groups using an MTS 858 Mini Bionix II instrument (Figure 2g). Static axial compression and dynamic fatigue tests were performed on separate specimen sets. Specimens loaded to failure in the static tests were not reused for dynamic testing; dynamic loading was applied to independent constructs that had not undergone prior static-to-failure loading.

For static loading, a load was applied through a spherical bearing on the proximal surface of the bone, while the distal end of the specimen was fixed to a platform (Yang et al., 2018). An initial preload of 10 N was applied to each specimen, and then a compressive load was applied at a rate of 5 mm/min. The load was gradually increased until failure occurred in any component of the construct or the fracture site gap closed. Axial stiffness was calculated from the applied load and displacement data. Construct strength was determined by progressively increasing the load until failure. In addition, compressive loads of 25, 50, 75, 100 N were applied to groups 2b and 3b and the axial displacement of the actuator was recorded to validate the finite element models.

Dynamic cyclic loads were applied under force control with a sinusoidal loading of ranging 0 and 100 N at 1 Hz as a functional surrogate within a controlled comparative framework (Liu et al., 2021; Sobky et al., 2008). The selected peak load (100 N) should be interpreted as a reproducible laboratory surrogate rather than a direct replication of patient-specific, activity-dependent, multi-axial *in vivo* forearm loading. The fatigue testing procedure and failure criteria were conducted within a simplified and reproducible laboratory framework consistent with standardized plate fatigue evaluation principles (ASTM F382-24, 2024), to facilitate meaningful comparisons between fixation constructs. Fatigue failure was defined as plate or screw breakage, loosening of screws in the plate or bone, or permanent deformation resulting in a reduction in the fracture gap of over 5 mm. Loading was continued for 100,000 cycles or until the construct failed, whichever came first. The total number of cycles was chosen to represent 1,000 cycles per day over 3 months (Yang et al., 2018). Actuator displacement was recorded for comparison.

Since each group in the static axial compression test consisted of 5 specimens, the data were first checked for normality using the Shapiro-Wilk test. If the data met the assumption of normality, one-way Analysis of Variance (ANOVA) was used to compare the mean values of stiffness and strength between the groups. In cases where ANOVA indicated a significant difference, *post hoc* pairwise comparisons were performed using Tukey’s Honest Significant Difference (HSD) test to identify specific differences between groups. For groups that did not meet the assumption of normality, the non-parametric Kruskal-Wallis test was applied to compare the median stiffness and strength across the groups. If the Kruskal-Wallis test showed significant differences, *post hoc* pairwise comparisons were conducted using Dunn’s test to further evaluate group differences. All statistical analyses were performed using Statistical Package for the Social Sciences (SPSS version 20.0) and the significance level was set at p < 0.05.

### Finite element analysis

**FIGURE 3:**
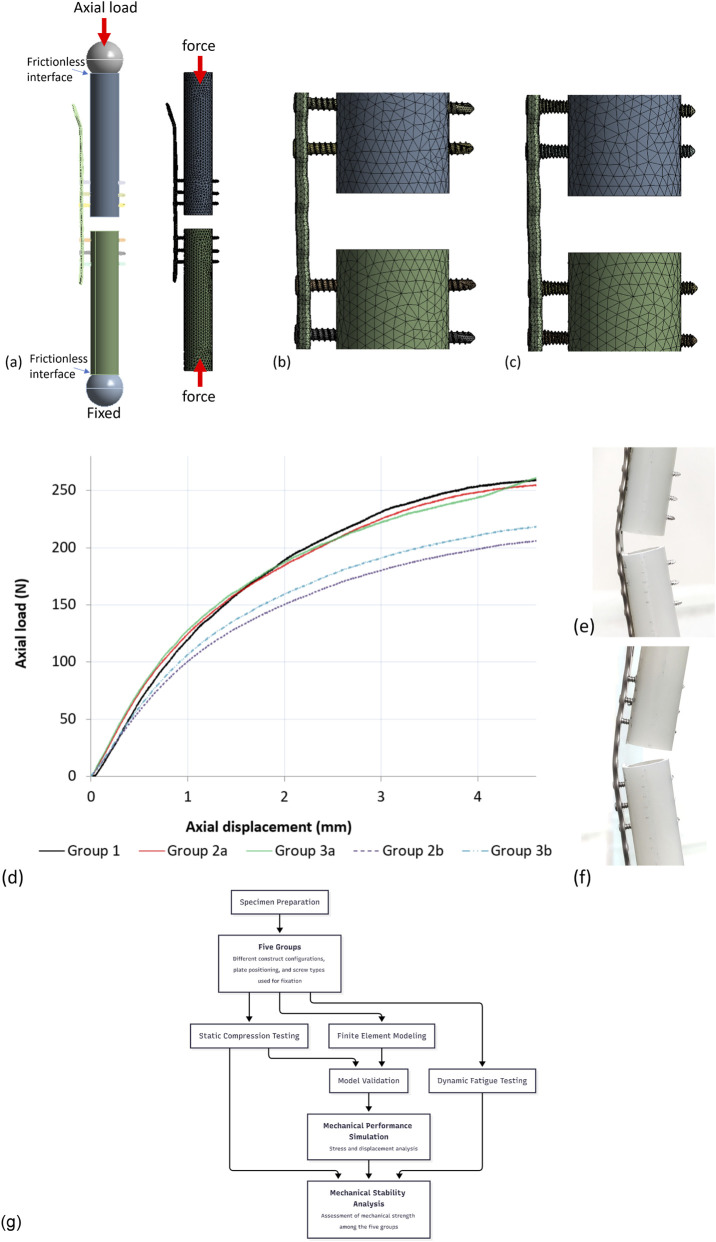
Meshed models and boundary conditions **(a)** showing the entire construct with a TDLR plate with FCL configuration, **(b)** a TDRL plate fixed with locking screws (group 2b), **(c)** a TDRL plate fixed with continuous locking screws (group 3b). **(d)** Load–deformation curves under static axial loading. **(e)** deformation of the bone plate with the plate positioned flush with the bone. **(f)** deformation of the bone plate with the plate offset from the bone. **(g)** Schematic overview of the experimental and finite element analysis workflow.

Five models, corresponding to the five groups for mechanical testing, were created in Creo Parametric 2.0 (PTC, MA, USA) and imported into Workbench 2021 (ANSYS, Inc., Canonsburg, PA, USA) for meshing and definition of boundary conditions. All solid components were meshed using tetrahedral elements (Figures 3a–c). Frictionless contact was applied to the interfaces between the cortical screw head/plate and plate/bone using surface-to-surface contact elements (CONTA174 and TARGE170). The interfaces between the bone screw/synthetic bone, locking screw head/plate, and continuous locking screw head/plate were defined as bonded. The synthetic bone was assigned a modulus of 15.8 GPa (Chong et al., 2007) and a Poisson’s ratio of 0.3 (Shih et al., 2023). The bone plate and bone screws were assigned a modulus of 110 GPa and a Poisson’s ratio of 0.3 (Shih et al., 2023).

Compressive loads of 25, 50, 75, 100 N were applied to a spherical bearing positioned on the upper cut surface of the proximal bone. These axial loads were used to validate construct-level response under standardized compression and were not intended to reproduce the full multi-axial physiological loading environment of the distal radius. The finite element models were validated by comparing the displacement results at the spherical bearing with those obtained from mechanical testing. The comparison revealed an error of less than 10% between the experimental models and finite element models.

A schematic overview summarizing the experimental design, mechanical testing procedures, and finite element analysis workflow is provided in Figure 3g.

## Results

### Results of mechanical tests

The static axial loading tests were conducted until construct failure (Figures 3d–f), and the results for Yield Load, Stiffness, and Ultimate Load are summarized in Table 2. For each parameter, the maximum and minimum values across the groups were identified.

**TABLE 2:** Static axial loading under load control until construct failure.

Mechanical parameter	Plate flush to bone			Plate far with bone	
	Group 1	Group 2a	Group 3a	Group 2b	Group 3b
Yield load (N)	126.00 ± 7.78	125.70 ± 7.72	127.34 ± 5.11	106.83 ± 7.52	107.73 ± 9.87
Stiffness (N/mm)	138.30 ± 6.49	155.78 ± 6.33	163.55 ± 7.92	119.39 ± 7.59	135.67 ± 6.84
Ultimate load (N)	253.41 ± 9.17	253.69 ± 5.54	259.70 ± 10.72	205.37 ± 10.54	204.81 ± 7.95

**FIGURE 4:**
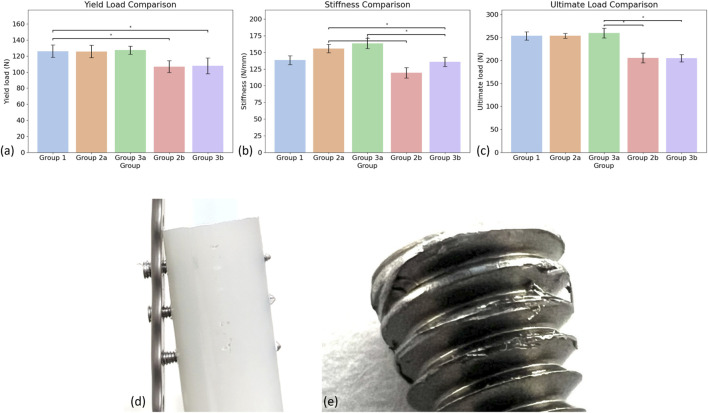
Comparison of mechanical performance across the five test groups. Bar plots illustrate the mean ± standard deviation for **(a)** Yield Load, **(b)** Stiffness, and **(c)** Ultimate Load. Statistically significant differences between groups were determined using one-way ANOVA followed by Tukey’s HSD test (p < 0.05). Significant pairwise differences are indicated with an asterisks (*). **(d)** Screw pull-out in Group 3b during the dynamic test; **(e)** Fatigue failure occurred on the threads of the continuous locking screw.

The yield load was highest in Group 3a (plate flush to bone) with a mean value of 127.34 ± 5.11 N, while the lowest was observed in Group 2b (plate offset from bone) at 106.83 ± 7.52 N. The Shapiro–Wilk test confirmed normality for the yield load data across all groups (p > 0.05), and one-way ANOVA revealed a statistically significant difference among the groups (p < 0.05). Post-hoc analysis using Tukey’s HSD test indicated that Group 3a had a significantly higher yield load than Groups 2b and 3b (p < 0.05), while no significant differences were found among Groups 1, 2a, and 3a (Figure 4a).

For construct stiffness, Group 3a exhibited the highest mean value (163.55 ± 7.92 N/mm), whereas Group 2b had the lowest (119.39 ± 7.59 N/mm). Normality was met for all groups, and one-way ANOVA demonstrated significant differences in stiffness (p < 0.001). Tukey’s HSD test revealed that Group 3a was significantly stiffer than Groups 2b and 3b, and Group 2a was significantly stiffer than Groups 2b and 3b (p < 0.01). Additionally, Group 1 had a significantly greater stiffness than Groups 2b and 3b (p < 0.01). No significant differences were found between Groups 1, 2a, and 3a (Figure 4b).

The ultimate load was the highest in Group 3a (259.70 ± 10.72 N) and the lowest in Group 3b (204.81 ± 7.95 N). Normality was satisfied, and one-way ANOVA showed a significant difference between the five groups (p < 0.001). Tukey’s HSD test confirmed that Groups 2b and 3b had significantly lower ultimate loads than Groups 1, 2a, and 3a (p < 0.001), while no significant differences were detected among the flush-to-bone groups (Figure 4c).

Dynamic cyclic loading tests were performed on all groups using a 100 N axial load and failure was defined as plate or screw breakage, loosening of screws in the plate or bone, or permanent deformation resulting in a reduction in the fracture gap of over 5 mm (Table 3). Group 2a demonstrated the highest fatigue resistance by enduring the greatest number of cycles before failure. In contrast, Group 3b had the lowest endurance, consistently failing early due to screw pull-out, indicating a distinct mechanical failure pattern (Figures 4d,e). Group 1 and Group 2b showed moderate endurance, with Group 1 sharing the same failure mode as Group 2a, while Group 2b failed under similar mechanisms but with markedly lower fatigue performance. Group 3a produced intermediate results, but unlike Groups 1 and 2, the construct failed due to screw pull-out (Figures 4d,e). These results highlight substantial inter-group variability, particularly between Groups 2a and 3b, underscoring the influence of implant design and fixation method on fatigue behaviour.

**TABLE 3:** Number of cycles endured under a 100 N axial load until failure, along with associated failure modes, across different experimental groups.

Group	Cycles under 100 N axial loading	Mean (range)	Failure mode
Group 1	83,164	81,500 (73,184–88,153)	Gap loss larger than 5 mm; bone plate deformed
	88,153		
	73,184		
Group 2a	100,531	101,201 (93,493–109,578)	Gap loss larger than 5 mm; bone plate deformed
	109,578		
	93,493		
Group 3a	74,357	76,900 (72,792–83,552)	Screw pull out
	83,552		
	72,792		
Group 2b	21,988	22,720 (20,448–25,725)	Gap loss larger than 5 mm; bone plate deformed
	25,725		
	20,448		
Group 3b	21,052	20,024 (18,883–21,052)	Screw pull out
	18,883		
	20,136		

### Results of finite element analysis

#### Validation result

**FIGURE 5:**
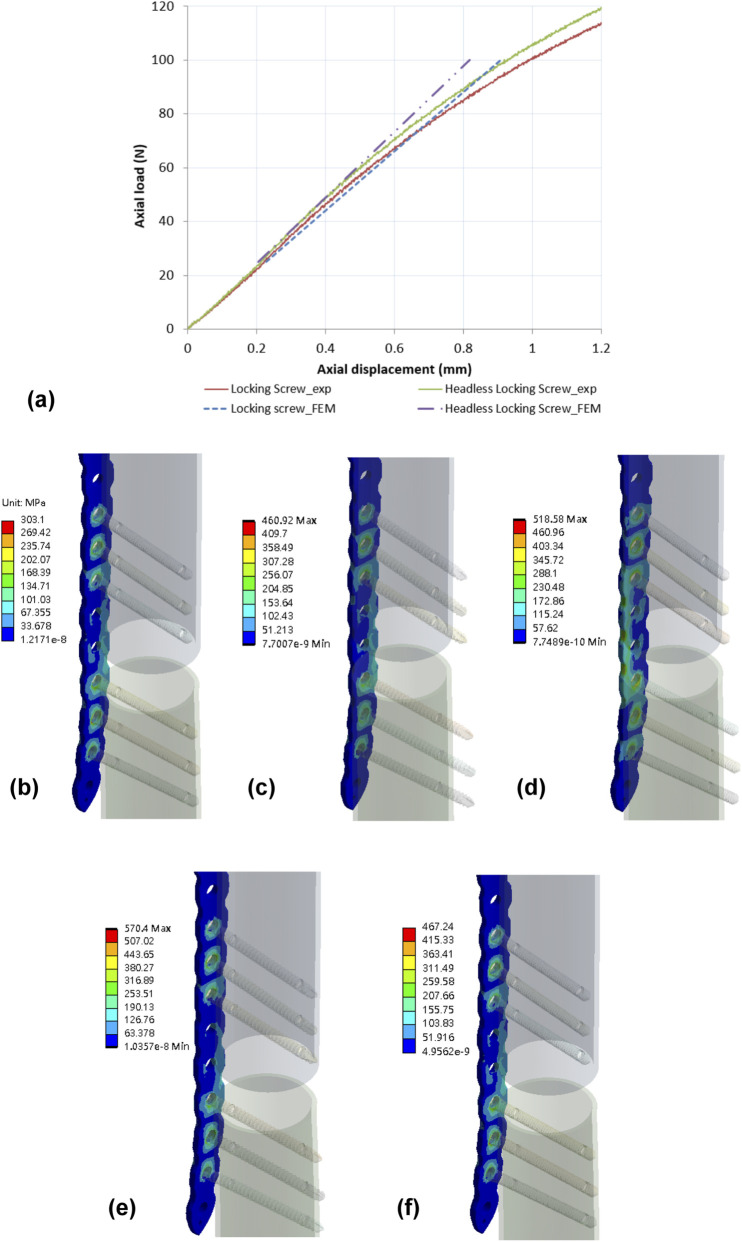
**(a)** Comparison of axial displacement between mechanical testing (exp) and finite element models (FEM) showed an error of less than 10% for both the locking screw and the continuous locking screw. Von Mises stress distribution on bone plates for **(b)** Group 1, **(c)** Group 2, **(d)** Group 3, **(e)** Group 4, and **(f)** Group 5.

The axial displacement of the spherical bearing recorded from the mechanical testing was compared against the data from the finite element models. Figure 5a shows the results of mechanical testing (exp) compared to the finite element models (FEM), with the error percentage of axial displacement being less than 10% with both the locking screw and the continuous locking screw.

#### Stress vand displacement on the models

**FIGURE 6:**
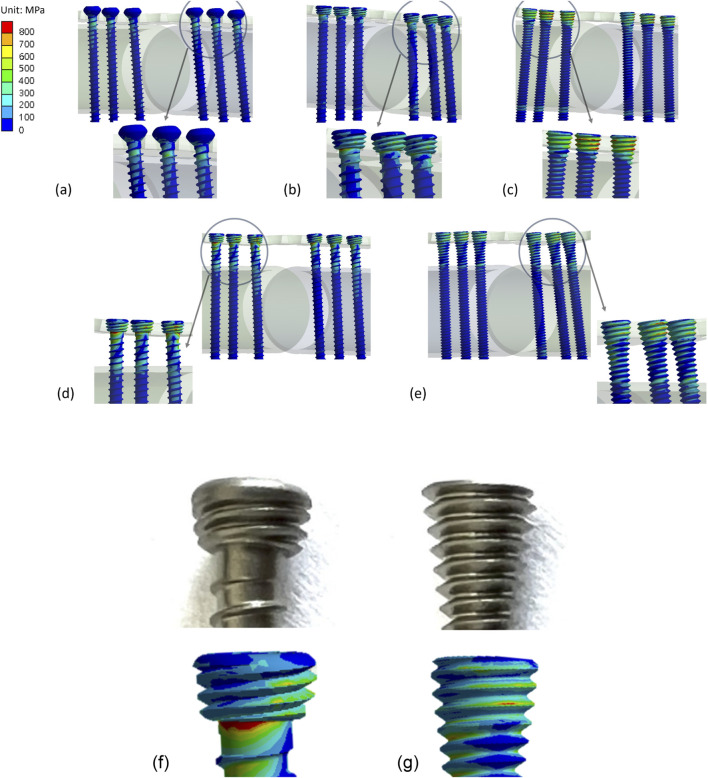
Von Mises stress distribution on bone screws for **(a)** Group 1, **(b)** Group 2, **(c)** Group 3, **(d)** Group 4, and **(e)** Group 5. Thread geometry of the **(f)** traditional locking screw and **(g)** continuous locking screw.

The maximum stress values at the plate and screw were recorded for each group in Table 4. For Group 1 (flush with bone), the maximum stress on the plate was 303.1 MPa and on the screw was 594.1 MPa (Figures 5b–f), with a higher concentration of stress observed at the neck region of the cortical screw and on the threads of the locking screw and continuous locking screw (Figures 6a–e).

**TABLE 4:** Maximum stress for each model and displacement of the spherical bearing under a 100 N axial loading.

Type of construct	Maximum stress (MPa)		Maximum displacement (mm)
Group	At plate	At screw	Axial loading direction
Group 1 (flush with bone)	303.1	594.1	0.77
Group 2a (Flush with bone)	460.92	973.5	0.62
Group 2b [with far cortical bone (4 mm)]	570.4	1,148.73	1.01
Group 3a (Flush with bone)	518.58	1046.94	0.70
Group 3b [with far cortical bone (4 mm)]	467.24	1,334.2	0.91

For Group 2a (flush), the maximum stress on the plate was 460.92 MPa, while the locking screws had a significantly greater stress of 973.0 MPa. The maximum stress on the plate for Group 2b (offset) was 570.4 MPa, but reached 1,148.73 MPa around the head region of the screw (Figures 6b,d).

In Group 3a (flush), the maximum stress on the plate was 518.58 MPa, while the maximum stress on the screw was found at the region where it contacted the bone plate and reached 1046.94 MPa. Group 3b (offset) had a maximum stress on the plate of 467.24 MPa and a stress of 1,334.2 MPa on the screw where it attached to the bone plate.

Table 4 also shows the axial displacement of each model. The greatest displacement occurred in Group 2b (1.01 mm), followed by Group 3b (0.91 mm). Group 1 exhibited the lowest displacement (0.77 mm), which was consistent with the relatively lower stresses observed with this configuration. Group 3a had a displacement of 0.70 mm, and the lowest value was recorded in Group 2a with a displacement of 0.62 mm.

## Discussion

Traditionally, cortical screws are not considered suitable for extra-periosteal plating because of the instability between the plate and screw. More recently, however, the development of locking plates has allowed for the screws to lock into the plate, providing angular stability to the screws after insertion. Nevertheless, it is worth noting that extra-periosteal fixation creates a longer lever arm for the locking screw head, which can increase the risk of breakage. A damaged screw head can make removal considerably more difficult (Gopinathan et al., 2013; Iwata et al., 2017). Our study found that, although the use of continuous locking screws resulted in a slightly greater structural stiffness (no significant difference) under static axial loading than standard locking screws, the thread design of the continuous locking screw caused an early pullout from the bone plate under dynamic conditions. With the A-Plus locking plate system, the results of this study did not show that the continuous locking screws could offer superior mechanical performance to traditional locking screw.

For the static compression test, all models failed due to bone plate deformation. No screw failures were recorded with either the locking screw or continuous locking screw. With the plate positioned flush to the bone, the results of the mechanical compression test did not show any significant differences between the three screw types. However, offsetting the plate from the bone significantly reduced the construct strength due to the longer lever arm (Ahmad et al., 2007; Yang et al., 2018). Considering Group 1 secured with traditional cortical screws as the control group, the results showed that offsetting the bone plate by 4 mm resulted in a significant drop in mechanical performance, regardless if the plate was secured with locking screws or continuous locking screws. The mechanical performance and failure modes were similar between the locking screw and continuous locking screw constructs under static compression loading. FCL was proposed as a dynamization strategy to reduce the stiffness of locked plating constructs while maintaining construct strength and has since been evaluated across multiple biomechanical studies (Bottlang et al., 2009; Bottlang et al., 2009; Deng et al., 2022; Kritsaneephaiboon et al., 2025). In the present study, the cyclic axial loading envelope (0–100 N) was selected as a standardized and reproducible laboratory surrogate for construct-level comparison rather than a direct replication of patient-specific, multi-axial *in vivo* forearm loading; axial loading on the order of ∼100 N has been used in distal radius fixation biomechanics and simulation frameworks for reproducible comparative evaluation (Liu et al., 2021; Sobky et al., 2008). As an additional context for upper-limb axial loading magnitude, load transmission has also been quantified under 100 N axial loading in cadaveric elbow biomechanics (Smithson et al., 2020).

For the dynamic compression test, the construct secured with continuous locking screws had a lower fatigue strength than that secured with traditional locking screws, both when the plate was positioned flush with the bone and offset from the bone. The continuous locking screws failed by screw back-out, indicating a failure of the locking mechanism, while none of the traditional locking screws failed by screw back-out in either test condition. The continuous locking screws showed obvious signs of thread damage, which may be caused by stress concentrations where the screw locks into the plate, possibly due to poor thread design. The locking thread pitch of the continuous locking screw differed from that of the traditional locking screw by 0.1 mm, and the taper angle of the screw head was smaller than that of the traditional locking screw (Figures 6f,g). From a mechanical standpoint, even a small mismatch in thread pitch or taper geometry can reduce the effective thread engagement area between the screw head and the plate hole. This geometric mismatch may introduce interfacial gaps that permit micromotion at the locking interface under cyclic loading. Such micromotion can amplify local stress concentrations on the locking threads, accelerating thread damage and ultimately leading to screw loosening or pull-out from the bone plate.

The FEM models showed von Mises stresses concentrated around the head of the continuous locking screw, while the traditional locking screw showed von Mises stresses at the transition area between screw head and shaft. Notably, the von Mises stress on the continuous locking screws was higher than on the traditional locking screw, which may explain why the screw backed-out during the dynamic axial bending test. Although the shape of the continuous locking screw could reduce the stress concentrations on the screw neck (Figures 6d,e), the stress was transferred to the locking thread on the screw head, causing damage to the thread. In this study, all materials in the finite element models were assumed to be homogeneous, isotropic, and linearly elastic. This simplification does not fully represent the heterogeneous and anisotropic nature of biological bone tissue and may therefore limit the accuracy of predicting localized microstructural stress behavior. However, the primary objective of the present finite element analysis was not to reproduce exact physiological stress states, but to enable a consistent, construct-level comparison of stress distribution among different screw–plate fixation configurations. Because all models were subjected to identical boundary conditions and material assumptions, the relative differences observed between constructs remain meaningful and directly comparable. This modeling approach is widely adopted in orthopaedic implant biomechanics when the focus is on comparative performance rather than absolute *in vivo* stress prediction.

While continuous locking screws were developed to improve stress distribution by eliminating abrupt geometric transitions at the screw head–neck junction, the present results indicate that their locking mechanism may be compromised in FCL configurations. In these conditions, improper thread matching between the screw head and the plate hole resulted in elevated stress concentrations on the locking threads, increasing the risk of thread damage and construct failure under cyclic loading. From a clinical perspective, continuous locking screws may be considered only when the plate is positioned flush with the bone and when limited cyclic loading is expected. However, in FCL constructs or situations requiring high fatigue resistance, traditional locking screws demonstrated more reliable fixation and superior resistance to mechanical failure. Furthermore, the increased number of shank threads in continuous locking screws may prolong insertion time, which could reduce surgical efficiency. Based on these findings, conventional locking screws remain the most appropriate choice for routine plate fixation in fracture treatment. Future design modifications that preserve the continuous head–neck geometry while adopting the thread and taper characteristics of traditional locking screws may be required before continuous locking screws can be recommended for broader clinical use.

There are some limitations to this study. First, although static and cyclic axial loading were performed using a standardized laboratory protocol, the loading conditions remain a simplified, uniaxial surrogate intended for construct-level comparison. The selected load magnitudes are experimentally tractable and physiologically plausible for laboratory evaluation; however, they do not replicate patient-specific, activity-dependent, multi-axial *in vivo* loading at the wrist/forearm. Therefore, the present findings should be interpreted as pre-clinical biomechanical evidence and cannot substitute for clinical outcomes. Second, the experimental setup and loading conditions were referenced from ASTM standards and do not accurately reflect actual clinical applications. The fatigue loading conditions used in this study represent a standardized laboratory model derived from static mechanical performance and are therefore not intended to directly replicate the magnitude or complexity of *in vivo* loading conditions acting on the forearm or wrist during daily activities. However, using an established standardised approach to testing allows the results from different screw types to be directly compared, which was the intent of this study. Similarly, to simplify the finite element models, the material properties were defined as homogeneous and isotropic. Finally, this study only considered a specific kind of locking plate system. Future studies may consider different designs for the plate shape and locking mechanism and altering the direction of loading.

## Conclusion

In conclusion, continuous locking screws placed flush with the bone demonstrated superior yield strength compared to far cortical locking (FCL) (4 mm offset) configurations. With an FCL configuration, the continuous locking screw showed lower stress concentrations at the screw neck, but transferred the stress to the screw thread where it engages with the plate hole, resulting in thread damage. The continuous locking screw design did not improve the mechanical strength of the construct in FCL configurations. These findings suggest that, while continuous locking screws offer mechanical advantages under certain conditions, such as when flush with the bone, further improvements to the thread design are necessary for continuous locking screws to be effective in FCL applications. In addition, because the loading protocol represents a standardized *in vitro* surrogate, further validation under more physiologically representative multi-axial loading and clinical studies is required before clinical recommendations can be made.

## Data Availability

The original contributions presented in the study are included in the article/Supplementary Material, further inquiries can be directed to the corresponding author.
