# Supplementary material for: Are continuous locking headless bone screws with locking plate fixation effective for bridging fracture gaps in far cortical locking constructs? An in vitro biomechanical and finite element study under simplified axial loading
Source: Front Bioeng Biotechnol. 2026 Mar 16;14:1736372. doi: 10.3389/fbioe.2026.1736372 (PMC13033729; doi:10.3389/fbioe.2026.1736372)
Supplement: Supplementary file 1 [file Supplementaryfile1.docx]

Annex:

Purpose:

This Annex provides: (i) a **material-assumption sensitivity analysis** to quantify how the choice of **homogeneous isotropic** versus **orthotropic** cortical bone properties affects key finite element (FE) outcome metrics; and (ii) a **quantitative stress-concentration comparison** between the **continuous locking screw** geometry and a **conventional locking screw** design to evaluate the stress redistribution within the screw–plate–bone construct under cyclic loading.

Methods:

### (i) Material-assumption sensitivity analysis (quasi-static evaluation across the cyclic loading envelope)

A validated finite element framework (main manuscript) was used to compute stress responses under standardized axial compression loading. Construct geometry, contacts, and boundary conditions were kept identical across analyses. To relate the FE evaluation to the experimental **cyclic axial loading protocol (0–100 N, 1 Hz)**, the model was evaluated at incremental load steps of **25, 50, 75, and 100 N**, spanning the cyclic loading envelope. These load steps were treated as **quasi-static snapshots** of the cycle and were used to quantify the effect of isotropic versus orthotropic bone properties across increasing load magnitude.

This quasi-static approach is appropriate for upper-limb loading because functional wrist motion during activities of daily living is predominantly low-frequency, with a predominant frequency component on the order of **~1 Hz** (Mann et al., 1989). Therefore, inertial amplification is expected to be limited, and construct-level stress comparisons can be reasonably represented using quasi-static snapshots of the cyclic loading envelope.

Two material assumption groups were analyzed (Table A). **Group A (Table A)** represents the **Sawbones cortical long-bone surrogate** used in the mechanical experiments and was modeled as **homogeneous, isotropic, and linearly elastic** (E = 15.8 GPa; ν = 0.3). **Group B (Table A)** used **orthotropic** material properties for human forearm cortical bone reported in a published forearm FE model (Coates et al., 2012), providing a physiologically representative comparison set for sensitivity analysis.

Table A. Material properties used in the finite element models for Group A (isotropic) and Group B (orthotropic)

|  | Group A | Group B (Coates et al., 2012) |
| --- | --- | --- |
| Young’s modulus (Cortical bone) | 15.8 (GPa) | E1 (GPa):16  E2 (GPa):6.9  E3 (GPa):6.3 |
| Shear modulus (Cortical bone) | 6.08 (GPa) | G_12_ (GPa):3.2  G_13_ (GPa):3.6  G_23_ (GPa):3.3 |
| Poisson's ratio (Cortical bone) | 0.3 | V_12_: 0.30  V_13_: 0.30  V_23_: 0.45 |
| Density, (Cortical bone) (kg/m^3^) | 1,640 | 1,900 |
| Young’s modulus  (bone plate and bone screws) | 110 GPa | 110 GPa |
| Poisson's ratio  (bone plate and bone screws) | 0.3 | 0.3 |
| Density, (bone plate and bone screws) (kg/m^3^) (Alegre et al., 2024) | 4,430 | 4,430 |

### (ii) Finite element analysis under dynamic cyclic loading (Transient Structural with inertia)

To quantitatively evaluate the effect of screw-head geometry on stress concentration and construct collaboration under **dynamic loading**, a **Transient Structural** analysis was performed for the plate–screw–bone construct, incorporating inertia through mass density. The same validated model geometry, contacts, and boundary conditions described in the main manuscript were used. Two screw designs were compared under identical conditions: (i) **conventional locking screws** and (ii) **continuous locking screws**, each evaluated in both **flush-to-bone** and **far cortical locking (FCL; 4 mm offset)** configurations (Groups 2a, 2b, 3a, and 3b; definitions as in the main manuscript).

#### Material models and density

All materials were modeled as **linear elastic**. The bone surrogate (hollow Sawbones cylinder) was assigned homogeneous isotropic properties consistent with the experimental surrogate (**E = 15.8 GPa**, **ν = 0.3**) and a density corresponding to the Sawbones cortical surrogate (ρ as defined in Table A). The plate and screws (Ti-6Al-4V) were assigned **E = 110 GPa**, **ν = 0.3**, and **ρ = 4430 kg/m³**.

#### Dynamic loading protocol (0–100 N, 1 Hz)

A sinusoidal cyclic axial force was applied via a spherical bearing on the proximal cut surface to replicate the experimental fatigue loading envelope. The load was prescribed as a time-dependent function:

$$F(t)=50\left( 1-\cos(2\pi ft) \right)\text{ N}$$

with $f=1$**Hz**, producing a smooth cyclic load ranging from **0 to 100 N** each cycle. The model was run for 5 cycles (5 s). Peak stresses were extracted from the final cycle to represent steady-cycle response. The analysis was run for multiple cycles to achieve repeatable peak response, and the distal boundary constraints were consistent with the experimental jig configuration (main manuscript).

Results:

(i) Material-assumption sensitivity analysis

A sensitivity analysis was performed using the **Group 3b configuration** (FCL offset 4 mm with continuous locking screws) to quantify the influence of bone material representation on key construct-level outcomes in the plate–screw–bone system. Consistent with the experimental **dynamic (cyclic) axial loading envelope (0–100 N)**, the FE model was evaluated at incremental load levels of **25, 50, 75, and 100 N**, spanning the cyclic loading range. Across all load steps, the orthotropic bone model (**Group B**) produced only minor differences relative to the isotropic baseline (**Group A**) in peak von Mises stress: peak stress at the **plate screw-hole region** decreased by ~**0.86%**, peak stress at the **screw-head region** changed by ~**0.24–0.25%**, and peak stress in the **bone near the screw hole** decreased by ~**4.21–4.22%** (Table B).

Importantly, the construct-level **axial displacement** in the loading direction also showed minimal sensitivity to the material assumption. The maximum displacement predicted by Group B was consistently slightly lower than Group A, with differences of approximately **−2.19% to −2.85%** across the evaluated load steps (e.g., **0.28 vs 0.274 mm at 25 N** and **0.91 vs 0.884 mm at 100 N**; Table B). Overall, the sensitivity analysis demonstrates that adopting **homogeneous isotropic** versus **orthotropic** cortical bone properties yields highly similar stress and displacement outcomes—particularly for the plate and screw—indicating that the isotropic bone assumption used in the main study does not materially influence the comparative conclusions under the applied loading range.

Table B. Sensitivity analysis of peak von Mises stress and displacement for Group 3b under axial compression (25–100 N)

|  | max. stress occurred at Plate (screw hole) | | | max. stress occurred at Screw head | | | max. stress occurred at Bone (screw hole) | | | Axial loading direction | | |
| --- | --- | --- | --- | --- | --- | --- | --- | --- | --- | --- | --- | --- |
|  | Max stress (MPa) | | | Max stress (MPa) | | | Max stress (MPa) | | | max. displacement (mm) | | |
| Load (N) | Group A | Group B | Δ% (B vs A) | Group A | Group B | Δ% (B vs A) | Group A | Group B | Δ% (B vs A) | Group A | Group B | Δ% (B vs A) |
| 25 | 116.81 | 115.80 | -0.864% | 333.55 | 332.73 | -0.247% | 36.11 | 34.59 | -4.213% | 0.28 | 0.274 | -2.190% |
| 50 | 233.63 | 231.61 | -0.866% | 667.09 | 665.48 | -0.241% | 72.23 | 69.18 | -4.218% | 0.49 | 0.478 | -2.480% |
| 75 | 350.44 | 347.41 | -0.865% | 1000.65 | 998.21 | -0.243% | 108.35 | 103.78 | -4.216% | 0.71 | 0.692 | -2.580% |
| 100 | 467.24 | 463.20 | -0.866% | 1334.20 | 1330.94 | -0.244% | 144.46 | 138.37 | -4.215% | 0.91 | 0.884 | -2.850% |

Model: Group 3b (FCL offset 4 mm) with continuous locking screws.

Group A: homogeneous, isotropic bone properties (baseline).

Group B: orthotropic bone properties (Coates et al., 2012).

(ii) peak and location-specific von Mises stresses at the screw, plate and bone comparison between the continuous locking screw geometry and the conventional locking screw design

Transient Structural analyses (including inertia via density) were performed under a **sinusoidal axial load (1 Hz, 0–100 N)**. Peak von Mises stresses were extracted at the **100 N peak-load phase** from three predefined hotspot regions: **plate (near screw hole)**, **screw (screw-head region)**, and **bone (near screw hole), as shown in Table C**.

Under the **flush-to-bone** configuration, the continuous locking screw construct (**Group 3a**) showed higher peak stresses in the implant than the conventional locking screw construct (**Group 2a**): plate stress increased from **469.22 MPa to 528.43 MPa** (**+12.6%**) and screw-head stress increased from **989.76 MPa to 1060.86 MPa** (**+7.2%**). In contrast, the peak stress in the surrounding bone decreased slightly (**178.99 MPa to 169.12 MPa; −5.5%**). These differences are consistent with the normalized ratios (Group 2a/Group 3a), where values <1 at the plate and screw indicate higher stresses for the continuous screw design, while the bone ratio >1 indicates lower bone stress for the continuous screw.

Under the **FCL offset (4 mm)** configuration, the stress redistribution pattern differed. Compared with the conventional locking screw construct (**Group 2b**), the continuous locking screw construct (**Group 3b**) reduced peak stress at the plate near the screw hole from **618.88 MPa to 475.18 MPa** (**−23.2%**) and slightly reduced screw-head stress from **1389.96 MPa to 1349.14 MPa** (**−2.9%**). However, peak bone stress near the screw hole increased from **132.89 MPa to 147.49 MPa** (**+11.0%**). The normalized ratios (Group 2b/Group 3b) reflect this shift: the plate ratio of **1.30** indicates substantially higher plate stress with conventional locking screws, whereas the bone ratio of **0.90** indicates higher bone stress with continuous locking screws under FCL as shown in Table D.

Overall, the transient cyclic analysis shows that **screw geometry and plate position interact** to govern where peak stresses localize within the screw–plate–bone system at the peak cyclic load.

**Table C. Maximum von Mises stress (MPa) under dynamic cyclic loading (1 Hz, peak load = 100 N).**

|  | Maximum Stress (MPa) at dynamic (1Hz) 100 N condition | | |
| --- | --- | --- | --- |
| Group | At plate (near screw hole) | At screw (screw head region) | At Bone (near screw hole) |
| Group 2a (Flush with bone) | 469.22 | 989.76 | 178.99 |
| Group 2b (with far cortical bone (4 mm)) | 618.88 | 1389.96 | 132.89 |
| Group 3a (Flush with bone) | 528.43 | 1060.86 | 169.12 |
| Group 3b (with far cortical bone (4 mm)) | 475.18 | 1349.14 | 147.49 |

Table D. Normalized maximum stress ratios comparing conventional versus continuous locking screw designs at the peak cyclic load (100 N).

| normalized max. stress | At plate (near screw hole) | At screw (screw head region) | At Bone (near screw hole) |
| --- | --- | --- | --- |
| Group 2a / Group 3a | 0.89 | 0.93 | 1.06 |
| Group 2b / Group 3b | 1.30 | 1.03 | 0.90 |

Reference:

Alegre, J. M., Díaz, A., García, R., Peral, L. B., Lorenzo-Bañuelos, M., & Cuesta, I. I. (2024). Mechanical and Fatigue Properties of Ti-6Al-4V Alloy Fabricated Using Binder Jetting Process and Subjected to Hot Isostatic Pressing. *Materials 2024, Vol. 17, Page 3825*, *17*(15), 3825. https://doi.org/10.3390/ma17153825

Coates, C., Goeser, P., Coates-Clark, C., & Jenkins, M. (2012). Impact response and simulation of damaged ulna with internal fixation. *Journal of Applied Biomechanics*, *28*(3), 324–334. https://doi.org/10.1123/jab.28.3.324

Mann, K. A., Wernere, F. W., & Palmer, A. K. (1989). Frequency spectrum analysis of wrist motion for activities of daily living. *Journal of Orthopaedic Research : Official Publication of the Orthopaedic Research Society*, *7*(2), 304–306. https://doi.org/10.1002/JOR.1100070219
